# Interplay of strain and intermixing effects on direct-bandgap optical transition in strained Ge-on-Si under thermal annealing

**DOI:** 10.1038/s41598-019-48032-4

**Published:** 2019-08-12

**Authors:** Chulwon Lee, Yang-Seok Yoo, Bugeun Ki, Min-Ho Jang, Seung-Hyuk Lim, Hyun Gyu Song, Jong-Hoi Cho, Jungwoo Oh, Yong-Hoon Cho

**Affiliations:** 1 Department of Physics and KI for the NanoCentury, Korea Advanced Institute of Science and Technology (KAIST), Daejeon, 34141 Republic of Korea; 2 School of Integrated Technology, Yonsei University, Incheon, 21983 Republic of Korea

**Keywords:** Semiconductor lasers, Optical materials and structures

## Abstract

The influence of thermal annealing on the properties of germanium grown on silicon (Ge-on-Si) has been investigated. Depth dependencies of strain and photoluminescence (PL) were compared for as-grown and annealed Ge-on-Si samples to investigate how intermixing affects the optical properties of Ge-on-Si. The tensile strain on thermally annealed Ge-on-Si increases at the deeper region, while the PL wavelength becomes shorter. This unexpected blue-shift is attributed to Si interdiffusion at the interface, which is confirmed by energy dispersive X-ray spectroscopy and micro-Raman experiments. Not only Γ- and L-valley emissions but also Δ_2_-valley related emission could be found from the PL spectra, showing a possibility of carrier escape from Γ valley. Temperature-dependent PL analysis reveals that the thermal activation energy of Γ-valley emission increases at the proximity of the Ge/Si interface. By comparing the PL peak energies and their activation energies, both SiGe intermixing and shallow defect levels are found to be responsible for the activation energy increase and consequent efficiency reduction at the Ge/Si interface. These results provide an in-depth understanding of the influence of strain and Si intermixing on the direct-bandgap optical transition in thermally annealed Ge-on-Si.

## Introduction

Over the past decade, germanium (Ge), a group IV semiconductor material, has become an attractive light source material for its potential application in Si compatible on-chip laser^1–7^. Owing to its high carrier mobility and compatibility with well-established silicon (Si) integrated circuit technology, it has been considered one of the most promising candidates for a light source monolithically integrated on Si for optical interconnection that can be directly combined with current complementary metal-oxide-semiconductor (CMOS) processes^4,8,9^. Although its indirect bandgap nature limits its emission efficiency, numerous studies aimed at realizing a Ge-based light source with improved efficiency have been reported by introducing a high level of tensile strain^1,6^ or a high Sn fraction GeSn alloy^10^ to achieve a direct bandgap. Strained Ge, in particular, can be easily realized by many different methods by using thermal annealing^11,12^, mechanical manipulation^2^, silicon nitride stressor layer^13^, or by forming micro- or nano-structures^1,6,14^. A remarkable achievement in optically pumped lasing was recently achieved in a highly strained Ge nanostructure fabricated on an insulator at low temperature^15^, by using wafer bonding process to avoid a defective region of Ge/Si interface. However, most of the recent attempts on strained Ge light source are based on Ge on insulator using wafer bonding process to avoid large threading dislocation density (TDD) at the Ge/Si interface^6,15^, or Ge grown on GaAs substrate^13^ to achieve high quality Ge film. Nevertheless, since the greatest benefit of using Ge is the Si compatibility, the monolithic integration of a Ge light source on Si is still remained as a holy grail.

Remaining obstacles on the monolithic Ge-on-Si research for the laser application are mostly associated with degraded crystal quality after epitaxial growth. Due to the large lattice mismatch between Ge and Si, a Ge layer on Si becomes defective and eventually may contain lots of non-radiative centers^3^. An easy and practical approach to reduce the TDD and to enhance tensile strain is adding cycles of post-growth thermal annealing^16,17^. Post growth annealing is also an essential method in the Ge-on-Si laser application for recovering crystalline and improving optical properties after high concentration n-type doping^18,19^. On the other hand, the post-growth annealing can induce intermixing of Si and Ge at the interface^9,20^, which is expected to change the energy levels of direct and indirect band edges, affecting not only the emission wavelength but also the thermal activation energy for the direct bandgap transition. Still, regardless of the practical importance of thermal annealing process on Ge-on-Si, there has been a lack of studies how the luminescence mechanism is affected by the thermal annealing process. Although Higashitarumizu *et al*. reported the influence of thermally-induced SiGe intermixing on the optical properties of n-type Ge-on-Si^21^, the activation energy perspective of SiGe intermixing has been rarely deliberated. Thus, a detailed understanding of such influences of thermal annealing on the Ge-on-Si is imperative for a realization of a monolithic Ge-on-Si device.

In this study, the influence of thermal annealing on the properties of Ge-on-Si has been investigated by structural and optical characterizations. To address how the thermal annealing and the consequent intermixing affects the luminescence property at the interface of strained Ge-on-Si, as-grown and thermally-annealed Ge-on-Si samples with different etched depths are compared. Not only the well-known L- and Γ-valley emissions but also an emission peak related to Δ_2_ valley could be found from our PL studies. From temperature-dependent PL (TDPL) studies, a significant change of thermal activation energies for the Γ- and Δ_2_-valley emissions was found after thermal annealing. The roles of thermally-induced strain and intermixing on the activation energy were concurrently considered to deduce the band structure change in Ge under the influence of thermal annealing. The degraded optical properties at the interfacial region of thermally annealed Ge-on-Si were explained by the band structure change, along with the contributions of defect-related levels and carrier escape to Δ_2_ valley.

## Results

### Influence of thermal annealing on strain

**Figure 1:**
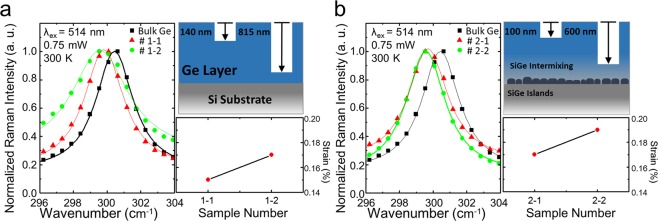
Schematic diagram of the experimental samples and measured strain values. (**a**) Raman spectra of as-grown samples and the strain value for different depth. (**b**) Raman Spectra and the strain values for the thermally annealed samples. The sample group with post-growth annealing shows significant overall enhancement of strain compared to as-grown samples.

To investigate the influence of thermal annealing on the optical and structural properties of Ge-on-Si, as-grown and thermally annealed Ge-on-Si samples were prepared. Supposing that the Ge layer is thermally influenced by Si interdiffusion, samples are etched with different depth to assess how different regions of the Ge layer are affected. From both as-grown and thermally annealed samples, two samples with shallow and deep etched depth are sorted. Samples are numbered with two digits, where the first stands for the presence of thermal annealing and the second for depth. For example, sample 1-2 is an as-grown sample with deeper etched depth whereas sample 2-1 is an annealed sample with shallow depth. The strain profile of each sample was investigated by micro-Raman experiments with a 514-nm laser excitation. Since the skin depth of a 514-nm laser is only a few tens of nanometers in Ge this may provide a more accurate strain profile depending on the etched depth from the surface. To eliminate local heating effect, zero power Raman shift was obtained by extrapolating power dependent Raman shift^22^. The power dependent Raman shift data can be seen from supplementary information. (Fig. S1) The tensile strain values plotted in Fig. 1 are calculated from the Raman shift of bulk Ge and other samples extracted at zero power. The coefficient used for the strain calculation is given by the recent result from Gassenq *et al*.^23^ Raman spectra from the annealed samples show a significant shift of the peak position, indicating strain enhancement by thermal annealing (Fig. 1). Note that the scale of the y-axis in the strain plot of Fig. 1a,b is the same. An overall enhancement of tensile strain can be observed from the samples after post-growth annealing, which agrees well with a previous report on the influence of cyclic thermal annealing on Ge-on-Si^17^. The as-grown samples showed different strain values for each depth, allowing PL measurement depending only on the value of strain without an influence of SiGe intermixing. Likewise, the thermally annealed samples showed different values of strain with different etched depth. Comparing the strain values from both groups of samples, an overall enhancement of tensile strain in the thermally annealed samples can be seen, in accordance with the previous literature^17^. The strain values from annealed samples are smaller than the value that can be obtained by considering the thermal expansion coefficient. Taking into account only the difference of thermal expansion coefficients between Si and Ge and assuming no strain relaxation during the cooling process, the thin film strain can be theoretically calculated as 0.29% with an annealing temperature of 800 °C and a cooling temperature of 25 °C^8^. The mismatch between the measured values and the theoretical value of thermally induced strain can be explained by the lattice mismatch between Si and Ge and strain-relaxation during the cooling process^8^. An atomic interdiffusion of Si may also compromise the strain at the proximity of Ge/Si interface^24^. Meanwhile, the measured strain value from the deeper region of the film has a larger value than that measured from the shallow region. This phenomenon can be intuitively understood by strain relaxation after depositing many thin layers. As the deposited layers are increased, the upper layer will be less influenced by the crystal structure and lattice mismatch at the interface. Therefore the strain at the near interface will be higher than the strain at the surface of the film^25^.

### SiGe intermixing at the interface

**Figure 2:**
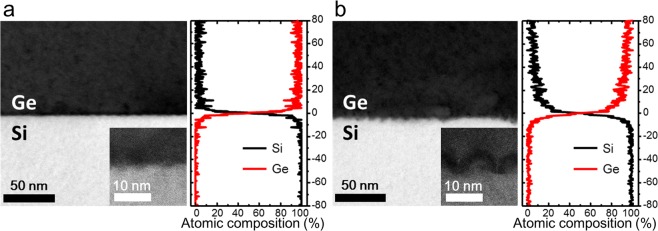
Comparison of structural and compositional change at the Si-Ge interface in terms of the presence of post-growth thermal annealing. (**a**) STEM and HRTEM (inset) image of the As-grown Ge-on-Si. Composition of Ge and Si atom measured by EDS mapping for the same region is shown on the right side of the STEM image. (**b**) After thermal annealing at 800 °C for 10 minutes.

Scanning transmission electron microscopy (STEM) were performed to study the influence of thermal annealing on the Si-Ge interface and how it affects the optical properties. Both as-grown and thermally annealed samples were measured and the cross-sections of their interface were compared. An abrupt boundary between the Si and Ge layers can be seen for the as-grown sample in the STEM image (Fig. 2a). There is no clear evidence of Si-Ge interdiffusion or SiGe alloy formation in the image. On the other hand, as seen in Fig. 2b, SiGe alloy island formation can be clearly identified from the interface of thermally annealed sample. A dark field STEM image and 2-dimensional electron dispersive x-ray spectroscopy (EDS) map in supplementary information also provide comprehensive evidences of SiGe alloy formation.(Fig. S3) A morphological difference can be also noticed from high resolution transmission electron microscopy images (HRTEM) in the inset of Fig. 2a,b. Unlike the interface of as-grown sample, the annealed sample is showing a significant morphology change at the interface due to the interdiffusion of Si atoms into the Ge layer. More transmission electron microscope images at the interface of each sample can be found in supplementary information (Fig. S1). To clarify the degree of SiGe interdiffusion at the interface, an EDS measurement was employed. EDS scanning was performed on the same region as that of the STEM images shown in Fig. 2. To view the atomic composition of Ge and Si at the interface, the K_A_ EDS intensity from Si and Ge for as-grown and thermally annealed samples is mapped. The scanning direction was perpendicular to the plane of the interface. Each data point indicates an integrated signal along the line parallel to the interface. Comparing the atomic composition profile of each sample, as shown in Fig. 2a,b, a significant difference between the as-grown and annealed samples can be recognized. As shown from the right-hand side of Fig. 2a, the as-grown sample shows an abruptly changing composition profile at the interface, indicating that there was no considerable SiGe intermixing from the growth and cooling process. On the other hand, the annealed sample shows a gradual composition change, indicating that thermal annealing induces SiGe intermixing, which can be seen from the right-hand side of Fig. 2b. It should be noted that the strain enhancement and Si interdiffusion are competing influences of thermal annealing. Although the thermal annealing induces tensile strain on Ge by thermal expansion mismatch, the thermally induced SiGe interdiffusion may generate a significant reduction of tensile strain at the interface^24^. Therefore, it is reasonable to deduce that the measured strain value at the proximity of the Ge/Si contains contributions of the both effects^24^.

### Interplay of strain and SiGe intermixing on photoluminescence property

**Figure 3:**
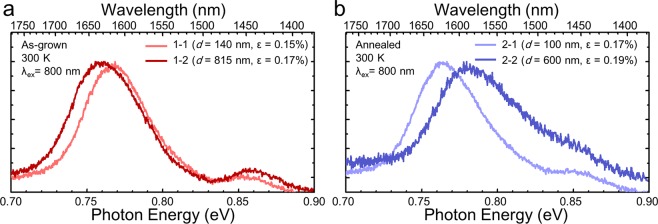
Comparison of room temperature PL spectra between As-grown samples and thermally annealed samples. (**a**) PL spectra from As-grown samples with different etching depth. Different etching depth gives rise to a strain difference. (**b**) PL spectra in the presence of thermal annealing.

In order to investigate the influence of thermal annealing on the luminescence properties of Ge-on-Si, PL measurements with an 800-nm laser excitation were carried out for the deep and shallow regions of the Ge film on a Si substrate. Supposing that the as-grown samples are not influenced by thermally induced SiGe intermixing, spectra from samples 1-1 and 1-2 are compared to exclude the intermixing effect on their optical properties. The assumption is also supported by our EDS measurement in Fig. 2a, which clearly shows that there has been no significant SiGe intermixing in samples 1-1 and 1-2. As it can be seen from Fig. 1a, samples 1-1 and 1-2 have different values of tensile strain. Strain induced direct bandgap transition^2,26^ at the spectral region of 0.73–0.80 eV changes with the value of tensile strain, as it is shown from Fig. 3a. It can be clearly seen that the spectrum from sample 1-2, which has a higher tensile strain than sample 1-1, is slightly red-shifted. Such strain induced red shift has been well described by deformation theory^2^. However, in the case of thermally annealed samples, the measured strain from sample 2-2 is higher than that of sample 2-1 (Fig. 1b), while the PL wavelength of sample 2-2 is shorter than that of sample 2-1, as shown in Fig. 3b. Comparing the spectra of sample 2-2 with sample 2-1, its PL peak wavelength is about 70 nm blue-shifted relative to that of sample 2-1. This blue-shift in PL spectrum could be attributed to Si-Ge intermixing, which was clearly observed from our EDS measurement. Since the thermal annealing process induces Si diffusion into the Ge layer, the optical properties of interfacial Si_x_Ge_1-x_ can be changed depending on the composition of Si^27^. Moreover, owing to the long skin depth of the 800 nm laser (~200 nm)^28^, which is almost 10 times longer than that of the 514 nm laser used for the Raman measurements, the PL spectra could contain more information from the Si mixed, deeper region of the Ge film. Accordingly, it is reasonable to conclude that the blue-shifted PL spectra from sample 2-2 is originated by Si interdiffusion at the proximity of Ge/Si interface.

It is worth pointing out that a peak at around 0.85 eV can be identified from all of the samples, as shown in Fig. 3a,b. The origin of the peak can be attributed to the emission from Δ_2_ valley. In the presence of tensile strain Δ valley splits into two valleys, namely Δ_2_ and Δ_4_ valley, and the Δ_2_ valley in particular, lies only 70 meV above the Γ valley^29^. The energy difference between Δ_2_ and the Γ-valleys is ~70 meV from sample 2-1 which matches the theoretical prediction by El Kurdi *et al*.^29^. Owing to the small energy difference between the Δ_2_ and Γ valleys, carriers in the Δ_2_ valley could be thermally excited from the Γ valley by phonon mediated processes. This can be supported by its temperature dependence similar to Γ valley transition, which will be discussed in the next section. In addition, PL energy of the Δ_2_ valley peak does not change substantially depending on the sample regardless of the significant blue-shift of the Γ valley. This also matches the theoretical prediction that the Δ valley of Si_x_Ge_1-x_ has only a small energy difference with the change of the Si composition^30^.

### Temperature dependent photoluminescence and influence of Si interdiffusion

**Figure 4:**
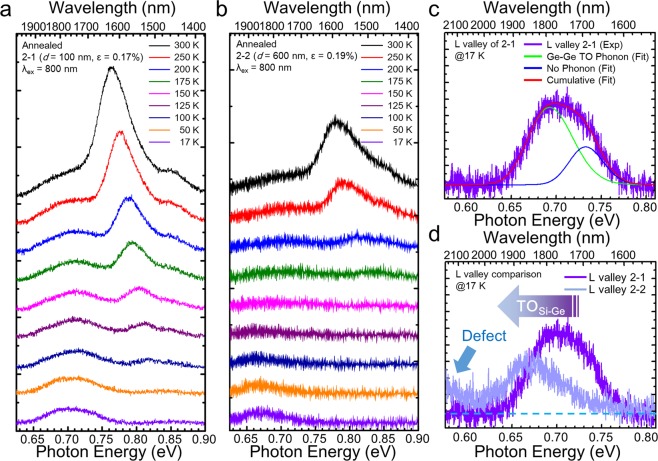
Temperature-dependent PL spectra from thermally annealed sample with different etched depths. (**a**) The sample with an etched depth of 100 nm. (**b**) The sample with an etched depth of 600 nm. (**c**) Double peak fitting result on the L valley spectrum of sample 2-1 at 17 K. (**d**) Comparison of L valley spectra at 17 K from both samples.

To study the influence of Si intermixing on the luminescence process in Ge, a TDPL experiment was employed. For the TDPL, samples 2-1 and 2-2 are measured for comparison with regard to the presence of SiGe intermixing. As sample 2-1 is assumed to be not affected by Si intermixing since the measured area is far from the Si-Ge interface, the TDPL result from sample 2-1 is expected to represent the temperature dependency of the optical properties from normal epitaxial strained Ge. On the other hand, as sample 2-2 was strongly affected by Si interdiffusion, the changes in its TDPL property can be attributed not only to the strain difference, but also to the presence of SiGe intermixing. For the Γ valley transition, as shown in the Fig. 4a,b, both samples 2-1 and 2-2 exhibit a continuous increase of the PL intensity with increasing temperature from 17 K to 300 K, which is consistent with a previous report by Sun *et al*., at a higher temperature range^31^. Since the Fermi-Dirac distribution allows an exponential increase of electron number in higher energy with increasing temperature, the number of electrons transferred from L valley to Γ valley increases exponentially and consequently Γ valley transition intensity increases with increasing temperature^31^. The intensity of the peak at around 0.85 eV, which is attributed to Δ_2_ valley emission, also increases as temperature increases, implying that the peak intensity is dominated by number of thermally populated electron in Δ_2_ valley, just as the intensity of Γ valley is. Unlike Γ and Δ_2_ valley transition, the intensity from L valley shows relatively small change with increasing temperature. This could be ascribed to an interplay of the decrease of electron population in L valley and the increase of phonon mediated radiative recombination with temperature.

The L valley emission at room temperature shows broad emission due to multi-phonon absorption and emission, while that at low temperature is dominated by phonon emission. As seen in Fig. 4c, the L valley spectrum of sample 2-1 consists of two peaks at 17 K. Our analysis resolved two distinct peaks centered at 0.693 eV and 0.733 eV, as shown in Fig. 4c, where the former can be attributed to a Ge-Ge transverse optical (TO) phonon emission peak with phonon energy of 40 meV and the latter can be attributed to a non-phonon L valley peak^32,33^. The energy difference from the fitting result is close to the reported Ge-Ge TO phonon energy value of 36 meV^32^. In the case of L-valley emission from sample 2-2, however, a peak having its energy of ~0.67 eV can be found at 17 K (Fig. 4b), which is lower than that of sample 2-1 at 17 K. (Fig. 4d) The origin of this peak can be attributed to the Si-Ge TO phonon emission induced by Si intermixing. Considering that the energy of Si-Ge TO phonon is given by 49 meV^32^, it is reasonable to conclude that the combined effect of the Si-Ge TO phonon emission and reduced phonon absorption at low temperature^34^ leads to the emergence of the peak at ~0.67 eV. Such Si-Ge TO phonon emission from SiGe was also reported from a thermally annealed SiGe heterostructure with low Ge content^35^.

**Figure 5:**
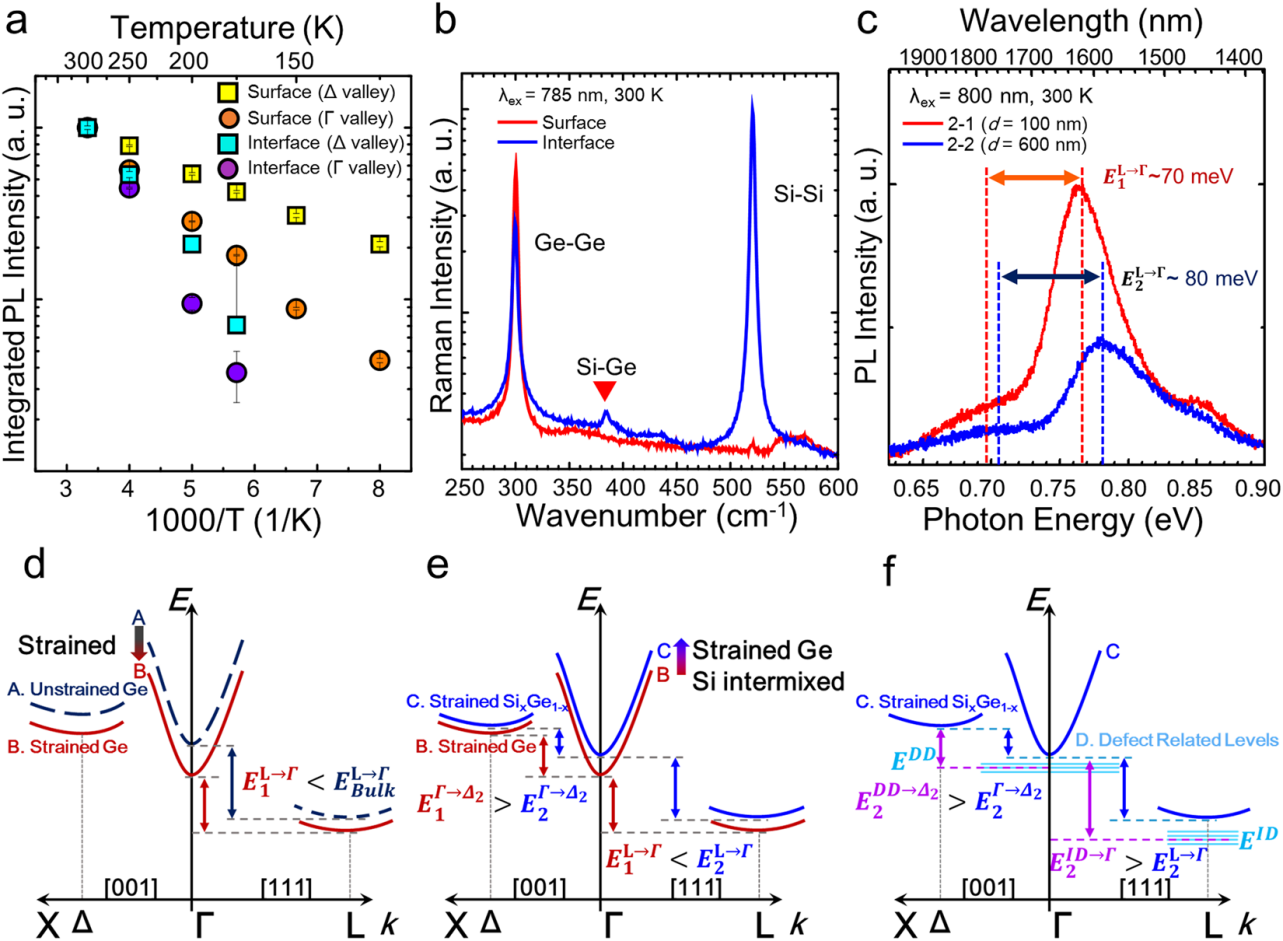
(**a**) Normalized intensity of Γ and Δ_2_ valley transition depending on the temperature for thermally annealed samples with a different region. A significant activation energy change could be found from the interface region of thermally annealed sample. (**b**) Comparison of Raman spectra from sample 2-1 and 2-2 under 785 nm laser excitation. (**c**) Room temperature PL spectra for the thermally annealed samples with different etched depth. (**d**–**f**) Illustration of conduction band changes under the influence of strain (**d**), Si intermixing (**e**), and in the presence of defect related levels (**f**). Note that Δ band splits into two bands Δ_2_ and Δ_4_ when tensile strained, and the lower energy band becomes Δ_2_.

The evidence of Si-Ge phonon mode could be also observed from a micro Raman measurement with 785 nm laser. Note that the wavelength of the laser was chosen to achieve a skin depth similar to that of the PL experiment. A significant peak of SiGe alloy mode could be found from sample 2-2, whereas no clear SiGe mode was be observed from sample 2-1. (Fig. 5b) The results from Raman measurement are in good agreement with the PL observation. It is noteworthy that no Si-Ge Raman peak was observed from the deeply etched as-grown sample (sample 1-2).

### Role of intermixing and defect on thermal activation energy

For the further study on the thermal activation energy change under the influence of Si interdiffusion, an Arrhenius analysis has been employed. The integrated PL intensity of the Γ-valley emission at 0.73–0.80 eV and the Δ_2_-valley emission at around 0.85 eV were extracted and plotted for each sample with respect to inverse temperature (Fig. 5a). By taking the data over the temperature range from 125 K (175 K) to 300 K into account for sample 2-1 (sample 2-2), the thermal activation energies for the Γ valley emission was extracted out by using Arrhenius relation, ${I}_{\Gamma }=A\,\exp (\,-\,{{\epsilon }}_{act}^{\Gamma }/kT)$, where ${{\epsilon }}_{act}^{\Gamma }={E}_{\Gamma }-{E}_{F}$, *A* is a proportional constant, and *k* is the Boltzmann constant^36,37^. Activation energy values could be calculated from a linear regression fitting of logarithm intensity of the Γ valley emission with respect to inverse temperature $[\,\mathrm{log}({I}_{\Gamma })=\,\mathrm{log}(A)-{{\epsilon }}_{act}^{\Gamma }/kT]$. Note that *E*_*F*_ is a quasi-Fermi level induced by optically pumping. A similar analysis was done for the Δ_2_-valley emission. Here, the energy difference between energy level *E*_1_ and *E*_2_ for sample 2-1 and sample 2-2 are denoted as ${E}_{1}^{{E}_{1}\to {E}_{2}}$ and ${E}_{2}^{{E}_{1}\to {E}_{2}}$, respectively, while the fitting values of thermal activation energy measured at *E*_2_ transition for sample 2-1 and 2-2 are indicated as ${{\epsilon }}_{1,act}^{{E}_{2}}$ and ${{\epsilon }}_{2,act}^{{E}_{2}}$, respectively. We found that ${{\epsilon }}_{1,act}^{\Gamma }$ = 58 meV and ${{\epsilon }}_{1.act}^{{{\rm{\Delta }}}_{2}}$ = 28 meV for sample 2-1, and ${{\epsilon }}_{2,act}^{\Gamma }$ = 122 meV and ${{\epsilon }}_{2,act}^{{\Gamma }_{2}}$ = 94 meV for sample 2-2, respectively. From the fitted values of activation energies, several important consequences of thermal annealing should be pointed out.

We firstly note that ${{\epsilon }}_{1,act}^{\Gamma } < {{\epsilon }}_{2,act}^{\Gamma }$. This can be mainly attributed to Si interdiffusion, since the energy shift of Γ valley toward higher energy is larger than that of L valley in Si_x_Ge_1-x_ with an increase of Si composition^30^ as described in Fig. 5e (B → C). Accordingly the energy difference between those two valleys for sample 2-1, where only thermally induced tensile strain is considered (Fig. 5d, A → B), is greater than that of sample 2-2, namely, ${E}_{2}^{L\to \Gamma } > {E}_{1}^{L\to \Gamma }$ (Fig. 5e). This interpretation is also consistent with the relatively big blue-shift of PL peak energy of Γ valley compared to that of L valley, as shown in Fig. 5c.

One may expect that ${{\epsilon }}_{1,act}^{{{\rm{\Delta }}}_{2}} > {{\epsilon }}_{2,act}^{{{\rm{\Delta }}}_{2}}$ since ${E}_{1}^{\Gamma \to {{\rm{\Delta }}}_{2}} > {E}_{2}^{\Gamma \to {{\rm{\Delta }}}_{2}}$ when only considering the larger energy shift of Γ valley than that of Δ_2_ valley in Si_x_Ge_1-x_ with increasing Si composition, as described in Fig. 5d^30^. However, interestingly, it is found that ${{\epsilon }}_{1,act}^{{{\rm{\Delta }}}_{2}} < {{\epsilon }}_{2,act}^{{{\rm{\Delta }}}_{2}}$ in our experiment. This mismatch can be explained by the presence of defect related levels in the Γ valley, as sample 2-2 is located at the proximity of Ge/Si interface with higher TDD. If quasi-Fermi level lies at a defect level in the direct valley (*E*_*DD*_) due to high defect density at the interface, more thermal energy will be required to excite photocarrier from *E*_*DD*_ into the Δ_2_ valley. Accordingly, the measured activation energy ${{\epsilon }}_{2,act}^{{{\rm{\Delta }}}_{2}}$ becomes ${E}_{2}^{DD\to {{\rm{\Delta }}}_{2}}$ instead of ${E}_{2}^{\Gamma \to {{\rm{\Delta }}}_{2}}$, as illustrated in the Fig. 5f (C→D). Such defect related level located at a lower energy than the Γ valley edge already has been reported^38^. Similarly, such defect related level can also explain ${{\epsilon }}_{2,act}^{\Gamma }$ (=122 meV) being larger than the PL energy difference of the Γ- and L-valley emission peaks (${E}_{2}^{L\to \Gamma }$~ 80 meV) as shown in Fig. 5c, where the energy difference was estimated by Gaussian fitting of each valley. Having a defect level in the indirect L valley (*E*_ID_) below the conduction band edge, a higher energy is required for a carrier activation from *E*_*ID*_ into the Γ valley. As a result, similar to the case of ${E}_{a2}^{DD\to {{\rm{\Delta }}}_{2}}$, the measured activation energy ${{\epsilon }}_{2,act}^{\Gamma }$ becomes ${E}_{2}^{ID\to {\rm{\Gamma }}}$ rather than ${E}_{2}^{L\to \Gamma }$.

In our case the defect related level luminescence can be noticed by a low energy signal arising at around 0.62 eV at 17 K for sample 2-2 (indicated by a pointing arrow in Fig. 4d). Although the whole spectrum could not be resolved due to the spectral limit of the detector, the small peak is clearly above the noise level (blue dotted line in Fig. 4d) of L valley spectra for both sample 2-1 and 2-2. This emission vanishes as the temperature increases probably due to thermal escape of the defect trapped carriers. Moreover, such emission at low energy could not be found from the sample 2-1, as shown in Fig. 4d, where the crystal quality is expected to be better than the sample 2-2. Thus, the increased activation energies in sample 2-2 can be explained by the presence of defect related level. (${{\epsilon }}_{2,act}^{\Gamma }\approx {E}_{2}^{ID\to \Gamma }$, ${{\epsilon }}_{2,act}^{{{\rm{\Delta }}}_{2}}\approx {E}_{2}^{DD\to {{\rm{\Delta }}}_{2}}$).

Finally, we also would like to note that ${{\epsilon }}_{1,act}^{\Gamma }$ < ${E}_{1}^{L\to \Gamma }$ (~70 meV), which contradicts to the previous reports^31,36^. This can be explained simply by an elevated quasi-Fermi level in L valley of sample 2-1 due to high power pulse laser excitation. Unlike the case of sample 2-1, however, ${{\epsilon }}_{2,act}^{\Gamma }$ $(\,\approx \,{E}_{2}^{ID\to {\rm{\Gamma }}})$ > ${E}_{2}^{{\rm{L}}\to {\rm{\Gamma }}}$, as elevated quasi-Fermi level is still residing at the defect related level ${E}^{ID}$.

## Discussion

Most of the previous researches have only focused on the beneficial aspect of post growth annealing such as strain enhancement and improved crystal quality^17,19,21^. As most of those studies have only observed PL spectra from surface of a region far from the Ge/Si interface, the luminescence information from the inside of Ge layer has been limited by the skin depth of lasers, and accordingly, the impact of Si interdiffusion on the activation energies has rarely been discussed. However, as annealing cycle or time increases Si interdiffusion extends further from the interface^24^, and consequently much wider range of Ge film on Si can be affected by SiGe intermixing. Our study could focus on the activation energy aspect by measuring the PL spectra from deeply etched region. From the activation energy comparison between sample 2-1 (close to surface) and sample 2-2 (close to Ge/Si interface), we found that ${{\epsilon }}_{act}^{\Gamma }$ is significantly increased at the proximity of Ge/Si interface by increased Si composition, and hence the carrier activation from L valley into Γ valley can be hindered by the increased ${E}^{L\to \Gamma }$^29^, This can be a major origin of the efficiency reduction of direct bandgap, Γ-valley transition at the Ge-Si intermixed region.

It is also worth noting that the thermal activation of carriers from Γ to Δ_2_ valleys also should be considered as a loss mechanism in strained Ge-on-Si. Moreover, Si intermixing increases the possibility of carrier activation from Γ to Δ_2_ valley, since ${E}^{\Gamma \to {{\rm{\Delta }}}_{2}}$ is known to be decreased by an increase of Si fraction^30,39^. Based on these facts, we may expect that SiGe intermixing effect (without considering the shallow defect levels mentioned earlier) can induce more carrier escape from Γ to Δ_2_ valleys and hence degrade optical efficiency of Γ-valley transition. As the efficiency reduction by Si interdiffusion (less Γ valley activation and more Δ_2_ valley escape) and the reduction of TDD are competing consequence of thermal annealing, the number cycle or time of the annealing process should be carefully decided to maximize the efficiency of Ge-on-Si based devices.

## Conclusion

We investigated the influence of post-growth thermal annealing on the optical properties of Ge-on-Si. The depth dependent strain profile was observed from thermally annealed Ge-on-Si by using micro-Raman scattering experiments. It turned out that the strain value is higher at the proximity of thermally annealed Ge/Si interface compared to the value measured at the surface of Ge film. The photon energy of the Γ valley PL peak, on the other hand, is higher at the interfacial region regardless of its higher strain, indicating the presence of Si interdiffusion. The SiGe intermixing could be confirmed by EDS mapping and Raman measurement. The activation energies for Γ-and Δ_2_-valley emissions and Δ_2_ valley could be derived from TDPL analysis. The carrier activation to Δ_2_ valley is suggested as a potential loss channel for the Γ-valley emission. A comparative TDPL study on shallow and deep regions of Ge-on-Si revealed that the activation energies for both Γ- and Δ_2_-valley emissions are increased at the proximity of Ge/Si interface. The increase of ${{\epsilon }}_{act}^{\Gamma }$ can be attributed to the formation of intermixed SiGe. It is suggested that the defect levels are also responsible for the increased activation energies based on the comparison between the measured data and theoretical results from previous literatures. As a result, we constructed a comprehensive energy band diagram of the Ge-on-Si system under the influence of strain, SiGe intermixing, and/or shallow defect levels. This shows how the interplay between these factors affects the direct and indirect bandgap transitions in the Ge-on-Si system, providing the important guide for the realization of monolithic Ge-on-Si devices with high efficiency and performance.

## Methods

### Sample fabrication

A Ge epitaxial layer with thickness of 1,000 nm was deposited on a Si (100) substrate by a reduced-pressure chemical vapor deposition (RPCVD) method. An influence of doping is excluded by using non-intentionally doped Ge samples for all the experiments. The prepared as-grown Ge sample is divided into two parts. One is remained and the other is annealed at 800 °C for 10 minutes in N_2_ ambient. As the post-growth annealing is expected to affect the strain as well as the Si fraction in the Ge layer, the samples are etched with different depth. The etching process was done by using 30% hydrogen peroxide (H_2_O_2_) solution as a wet-etchant, to systematically analyze the influence of thermal annealing on Ge-on-Si. By controlling the etching time, different etching depth on Ge-on-Si could be achieved, where the etching rate was given by 60~80 nm/min at the conditions we used. Since we could not achieve exactly the same etching conditions on each sample, etched samples with similar depth from as-grown and thermally annealed Ge-on-Si were sorted and compared. The sample numbering is given by a combination of two digits, where the first indicates the type of sample and the second indicates the depth. Namely, sample 1-2 indicates the sample without annealing with the deepest etched depth, while sample 2-1 indicates the thermally annealed sample with shallow depth. The sample numbering convention in this study and a schematic of the samples are presented in Fig. 1.

### Structural characterization

STEM measurements were performed by using Cs-Corrected Scanning Transmission Electron Microscopy by JEOL (JEM-ARM200F) and EDS spectra were achieved from Bruker Quantax 400.

### Optical characterization

A HORIBA micro-Raman spectroscopy system was employed to measure the strain values of the samples. Both 514 nm and 785 nm laser sources were used for the Raman measurements. For the PL measurements, a Chameleon Ti:sapphire femto-second laser with 180 mW average power was used with an Acton SP-300i monochromator. All the PL spectra were obtained under exactly the same experimental conditions for a comprehensive and comparable study with 800 nm excitation wavelength. The PL measurements were performed in a cryostat for a further TDPL experiment. The TDPL experiment was performed on samples 2-1 and 2-2 at the temperature region of 17 K to 300 K to achieve activation energy of each sample. All the TDPL measurements have been performed under exactly the same conditions including room-temperature measurements. The PL spectra are subjected to a least square fitting using Gaussian profile.

## Supplementary information

Supplementary Information for: Interplay of strain and intermixing effects on direct-bandgap optical transition in strained Ge-on-Si under thermal annealing
