# Supplementary material for: Interplay of strain and intermixing effects on direct-bandgap optical transition in strained Ge-on-Si under thermal annealing
Source: Sci Rep. 2019 Aug 12;9:11709. doi: 10.1038/s41598-019-48032-4 (PMC6690974; doi:10.1038/s41598-019-48032-4)
Supplement: Supplementary file 1 — Supplementary Information for: Interplay of strain and intermixing effects on direct-bandgap optical transition in strained Ge-on-Si under thermal annealing [file 41598_2019_48032_MOESM1_ESM.docx]

**Supplementary Information for:**

**Interplay of strain and intermixing effects on direct-bandgap optical transition in strained Ge-on-Si under thermal annealing**

**Chulwon Lee**^1^**, Yang-Seok Yoo**^1^**, Bugeun Ki**^2^**, Min-Ho Jang**^1^**, Seung-Hyuk Lim**^1^**, Hyun Gyu Song**^1^**, Jong-Hoi Cho**^1^**, Jungwoo Oh**^2^**, and Yong-Hoon Cho**^1^*****

^1^Department of Physics, Korea Advanced Institute of Science and Technology, Daejeon 34141, Republic of Korea

^2^School of Integrated Technology, Yonsei University, Incheon 21983, Republic of Korea

*Correspondence: Prof. Yong-Hoon Cho

Department of Physics, Korea Advanced Institute of Science and Technology, Daejeon 34141, Republic of Korea. Tel: (82) 42-350-2549, Email: [yhc@kaist.ac.kr](mailto:yhc@kaist.ac.kr)

**Figure S1 : Power dependent Raman measurement**

To eliminate the influence of laser heating on the Raman shift, we performed power dependent Raman experiment As Raman shift shows linear dependence on the excitation power, we extrapolate the y-axis intersection to achieve zero power Raman shift for each of the samples.


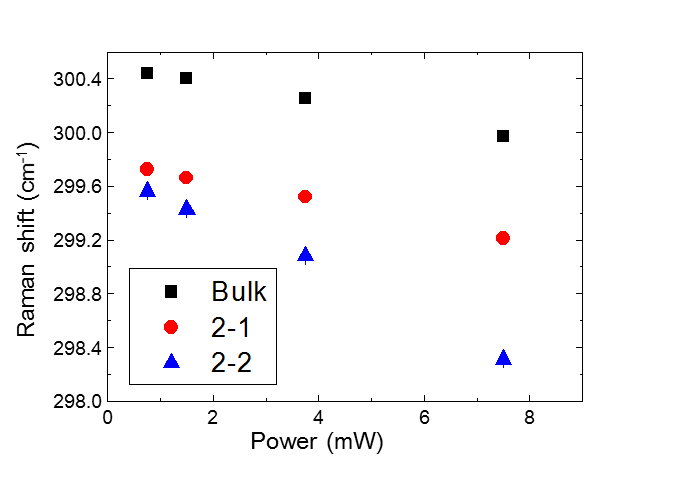


**Figure S1.** Power dependent Raman shift data for Bulk, sample 2-1, and sample 2-2. All the samples showed linear power dependence but with different slope. Intercept of the linear fitting of each data was taken to calculate zero power strain.

**Figure S2 : Transmission electron microscope measurement for threading dislocation observation.**


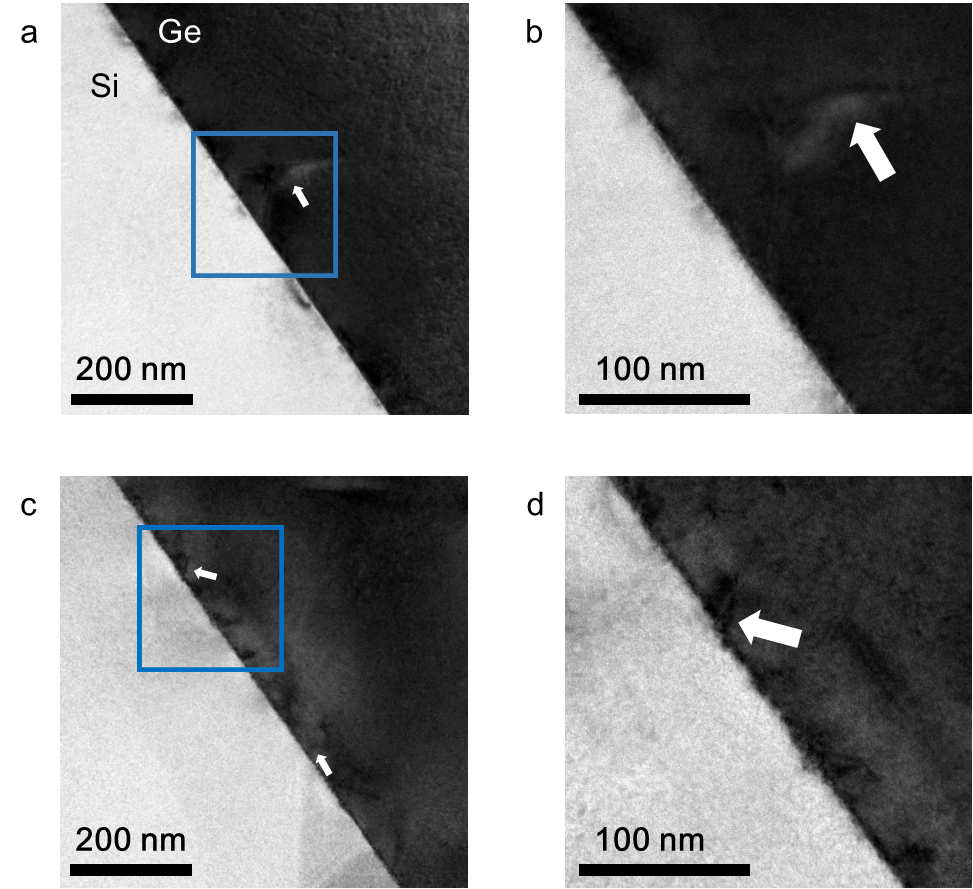


**Figure S2.** Comparison of TEM images at Ge/Si interface between as-grown (figure a and b), thermally annealed (figure c and d). (a) Threading dislocations are clearly identified at the interface of the as-grown sample (White pointing arrows). (b) Higher magnification image of the blue-squared area in figure (a). (c) Dislocations are confined at the Ge/Si interface of thermally annealed sample (White pointing arrows). (d) Higher magnification image of the blue-squared area in figure (c).

**Figure S3 : 2D Electron Dispersive X-ray Spectroscopy Mapping**


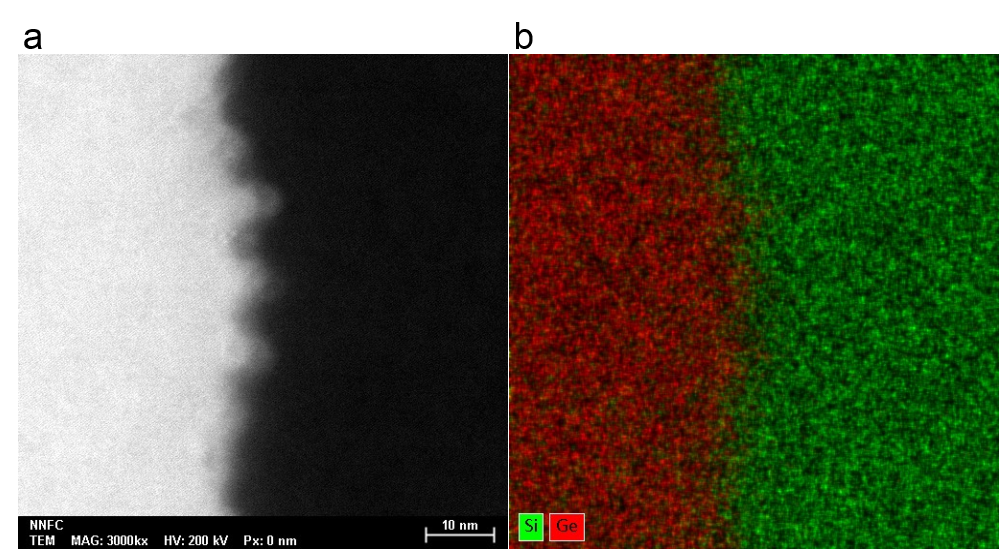


**Figure S3.** 2-Dimensional EDS mapping on the interface. (a) Dark field scanning transmission electron microscopic image at the interface of sample 2-2. (b) EDS mapping image at the same area of the (a). Clear SiGe intermixing can be observed.
